# Prevalence and magnitude of groundwater use by vegetation: a global stable isotope meta-analysis

**DOI:** 10.1038/srep44110

**Published:** 2017-03-10

**Authors:** Jaivime Evaristo, Jeffrey J. McDonnell

**Affiliations:** 1 Global Institute for Water Security and School of Environment and Sustainability, University of Saskatchewan, Saskatoon, Canada; 2 School of Geosciences, University of Aberdeen, Aberdeen, Scotland, United Kingdom; 3 Department of Forest Engineering, Resources and Management, Oregon State University, Corvallis, Oregon 97330 USA

## Abstract

The role of groundwater as a resource in sustaining terrestrial vegetation is widely recognized. But the global prevalence and magnitude of groundwater use by vegetation is unknown. Here we perform a meta-analysis of plant xylem water stable isotope (δ^2^H and δ^18^O, *n* = 7367) information from 138 published papers – representing 251 genera, and 414 species of angiosperms (*n* = 376) and gymnosperms (*n* = 38). We show that the prevalence of groundwater use by vegetation (defined as the number of samples out of a universe of plant samples reported to have groundwater contribution to xylem water) is 37% (95% confidence interval, 28–46%). This is across 162 sites and 12 terrestrial biomes (89% of heterogeneity explained; *Q-value* = 1235; *P* < 0.0001). However, the magnitude of groundwater source contribution to the xylem water mixture (defined as the proportion of groundwater contribution in xylem water) is limited to 23% (95% CI, 20–26%; 95% prediction interval, 3–77%). Spatial analysis shows that the magnitude of groundwater source contribution increases with aridity. Our results suggest that while groundwater influence is globally prevalent, its proportional contribution to the total terrestrial transpiration is limited.

Water uptake by vegetation and transpiration return about half of terrestrial precipitation to the global water cycle^1^. Partitioning of terrestrial precipitation inputs among transpiration, evaporation, soil water storage and groundwater recharge in different environments, however, remains poorly understood^2^. Quantifying the influence of groundwater on transpiration is important in part because of recent evidence suggesting widespread separation between groundwater and transpiration^3^,^4^, and because of the recognized relevance of groundwater to large-scale land surface processes^5^,^6^,^7^. While the percent uptake of groundwater by plants has been estimated at large scales using Geographic Information System-based methods^8^,^9^ and land surface model approaches^10^,^11^, only stable isotope techniques^12^ can provide direct means for tracking the water sources of plants^13^. Since root water uptake is generally a non-fractionating process^14^,^15^,^16^, the isotopic composition (δ^2^H and δ^18^O) of xylem (i.e. plant stem) water should reflect its sources within the rooting zone. This fundamental understanding of water uptake and transport from roots to shoots underlies the utility of stable isotopes in plant water uptake investigations^17^. While many site-based studies have now been completed^18^,^19^,^20^,^21^,^22^,^23^,^24^,^25^,^26^,^27^,^28^,^29^,^30^,^31^,^32^,^33^,^34^,^35^,^36^,^37^,^38^,^39^,^40^,^41^,^42^,^43^,^44^,^45^,^46^,^47^,^48^,^49^,^50^,^51^,^52^,^53^,^54^,^55^,^56^,^57^,^58^,^59^,^60^,^61^,^62^,^63^,^64^,^65^,^66^,^67^,^68^,^69^,^70^,^71^,^72^,^73^,^74^,^75^,^76^,^77^,^78^,^79^,^80^,^81^,^82^,^83^,^84^,^85^,^86^,^87^,^88^,^89^,^90^,^91^,^92^,^93^,^94^,^95^,^96^,^97^,^98^,^99^,^100^,^101^,^102^,^103^,^104^,^105^,^106^,^107^,^108^,^109^,^110^,^111^,^112^,^113^,^114^,^115^,^116^,^117^,^118^,^119^,^120^,^121^,^122^,^123^,^124^,^125^,^126^,^127^,^128^,^129^,^130^,^131^,^132^,^133^,^134^,^135^,^136^,^137^,^138^,^139^,^140^,^141^,^142^,^143^,^144^,^145^,^146^,^147^,^148^,^149^,^150^,^151^,^152^, a global synthesis of these data has not yet been made.

Here we employ a meta-analytic review of 138 published papers (hereafter ‘source papers’), representing 7,367 xylem water measurements from 251 genera and 414 species of angiosperms (*n* = 376) and gymnosperms (*n* = 38) in 162 sites across 12 terrestrial biomes worldwide. To make appropriate quantification of groundwater influence and address publication bias, our systematic review encompasses all physiographic settings. We define “groundwater” following the operational definitions in the respective source papers, which includes ‘water below permanent groundwater tables’ or ‘aquifers to springs’ and ‘groundwater reservoirs in bedrock cracks and fissures’. We quantify this broad groundwater influence on xylem water by calculating the prevalence and magnitude of groundwater use. We define prevalence as the number of samples out of a universe of plant samples reported to have groundwater contribution to xylem water. We define magnitude as the proportion of groundwater contribution reported in the xylem water mixture (see Methods).

To quantify prevalence of groundwater influence, we compute prevalence point estimates and 95% confidence intervals (CIs) using a random-effects model with the source paper as the unit of analysis (see Methods). Figure 1 shows that the global mean prevalence of groundwater influence is 37% (95% CI, 28–46%). There is substantial variability between studies from a low of 0.05% to a high of 99%, reflecting site- and species-level differences across studies. Almost 90% (*I*^2^ = 88.91; *Q-value* = 1235; *df* = 137; *P* < 0.0001) of the observed variance in prevalence estimates is real. That is, on a global scale the true prevalence estimates for each study would be only a little smaller (~11%) than the wide site-to-site variability shown in Fig. 1. The high value of *I*^2^ further suggests that on a global scale, the true mean prevalence of groundwater influence on plant transpiration is in the CI range of 28–48% for 95% of all possible meta-analysis cases. On a terrestrial biome basis (Fig. 1a), groundwater influence is most prevalent in deserts 69% (95% CI, 51–82%), tropical grasslands 69% (95% CI, 23–94%), and montane grasslands 66% (95% CI, 32–88%). Groundwater influence is least prevalent in tropical coniferous forests 1% (95% CI, 0–17%), boreal forests 4% (95% CI, 0–22%), and temperate coniferous forests 17% (95% CI, 8–32%). These biome-level estimates of mean prevalence reflect the variability between major habitat types (Fig. 1b; *Q-value* = 39.7; *df* = 11; *P* < 0.0001), underlining differences across study sites and species.

To quantify magnitude of groundwater influence, we compute magnitude point estimates and 95% CIs using a random-effects model with genus as the unit of analysis (see Methods). Figure 2 shows that the global mean magnitude of groundwater influence is 23% (95% CI, 20–26%). As with the meta-analysis on prevalence, there is substantial variability in magnitude of groundwater use between genera, from exclusively soil water (e.g. *Cupressus* sp. and *Vernonia* sp.) to almost exclusively groundwater (phreatophytes *Sarcobatus* sp. and *Ericameria* sp.). About 67% (*I*^2^ = 66.62; *Q-value* = 1375; *df* = 250; *P* < 0.0001) of the observed variance in magnitude estimates is real. The high value of *I*^2^ further suggests that on a global scale, the true mean magnitude of groundwater influence on plant transpiration is in the CI range of 20–26% for 95% of all possible meta-analysis cases. Calculating the 95% prediction interval (PI) results in a true magnitude for any single study in the range of 3–77%, reflecting variability due to site-, species-, and genus-level differences. We note that a meta-analysis using species as the unit of analysis yields similar overall mean magnitude and 95% CI estimates compared to genus as the unit of analysis. This suggests that taxonomic control on the magnitude of groundwater influence is as important at the genus level as it is at the species level.

Figure 2 shows a superposition of prevalence (bubble plot) and magnitude (error bar plot), illustrating that both measures of groundwater influence are similar in 155 of 251 genera. This reflects studies where prevalence is of the same degree as magnitude, which results from exclusive use of either soil water (i.e. vadose zone water) or groundwater. In 96 of 251 genera, prevalence is greater than magnitude. This reflects studies where plant water use is a mixture of various sources. We explore these effects by performing an analysis across different source identification approaches. Bayesian mixing model results (using SIAR^153^ and MixSIR^154^; *n* = 8) tend to fall outside the lower limit of overall 95% CI (20–26%), while IsoSource^155^ model results (*n* = 34) and 2,3-source mixing model results (*n* = 32) tend to approximate the overall CI (Fig. 2a). A direct inference approach (*n* = 59), defined as direct comparison of hydrogen or oxygen isotopic composition between xylem water and subsurface water sources, lies between the Bayesian source-mixture approaches, while the customized (i.e. unique to papers) mixing model approaches (*n* = 5) tend to fall outside the upper limit of overall CI. While these show the differences between mixing model approaches in quantifying the magnitude of groundwater influence (*Q-value* = 54.25; *df* = 4; *P* < 0.0001), the variability resulting from these differences are reflected in the overall prevalence and magnitude estimates and corresponding 95% CI. This suggests that mixing model differences are important, but at the aggregated scale of this meta-analysis, these differences converge to yield the overall magnitude estimate of 23% (95% CI, 20–26%).

We next grouped the meta-analysis data into angiosperms and gymnosperms. Figure 2b shows that angiosperms exhibit greater magnitude of groundwater influence at 24% (95% CI, 21–28%) than gymnosperms at 16% (95% CI, 10–22%) (*Q-value* = 5.72; *df* = 1; *P* = 0.01). The main conducting elements in angiosperms (called xylem vessels) allow for wider variability in element size and wall thicknesses^156^ than their conducting element counterparts in gymnosperms (called tracheids). Moreover, angiosperms have a greater number of parenchyma cells^157^ that are linked to improved hydraulic system efficiency after stressful conditions such as drought^158^. These xylem anatomical differences may explain our finding that angiosperms tend to have greater groundwater influence than gymnosperms.

Finally, we explore the physical controls on our magnitude findings. Several factors are known to affect groundwater influence on vegetation, from physiological^159^, water table and rooting depths^5^,^6^,^160^, and species^161^,^162^, to rainfall patterns^9^, groundwater extractions^8^ and changes in stream flow regimes^163^. We supplement the primary information available from the source papers with site-specific datasets (see Methods) on rooting depth, rooting zone water storage amount, water table depth, potential evapotranspiration and aridity index, precipitation amount, and streamflow amount. We then use a batch algorithm and a locally weighted linear smoother (self-organizing maps clustering technique) to quantify the multivariate controls on the magnitude of groundwater influence on xylem water and the spatial structure of the meta-analysis database (see Methods). Our analysis shows that the database falls into seven optimal clusters. Figure 3 shows how these clusters plot along a theoretical curve (the Budyko curve, ref. 164) of evaporative index as a function of dryness index. The Budyko curve predicts actual evapotranspiration (AET) by potential evapotranspiration (PET) based on a simple water balance (streamflow = precipitation – evapotranspiration).

The computed magnitude of groundwater influence is strongly negatively correlated with aridity index of the modeled clusters (Fig. 3 inset, *R*^*2*^ = 0.96). This suggests that the interactions between variables (e.g. rooting depth, moisture conditions, water table depth, etc.) within a cluster are strong enough to predict the magnitude of groundwater influence (Fig. 3 map inset). Exceptions are clusters 2, 3, and 7, as well as four sites in highly urbanized settings, that fall below the theoretical prediction. That these deviations are along the y-axis (AET/P in Fig. 3, see figure caption for definition of terms) suggest that in those locations vegetation dynamics could not be predicted from a simple water balance.

Overall, our results demonstrate that while groundwater influence on vegetation is prevalent (~37% globally), its magnitude may not be as significant as is increasingly being argued in the literature^5^. Transpiration has been estimated to comprise between 39 ± 10%^1^ and 48 ± 10%^4^ of total terrestrial precipitation. Without making any assumptions about plant-level (e.g. stomatal regulation^160^) controls on plant water use strategies, our finding that the magnitude of groundwater contribution to total xylem water mixture is limited to ~23% globally translates to an estimate of groundwater contribution to xylem as a fraction of total terrestrial precipitation between 9 and 11%. This is consistent with the 11 ± 8% estimate of ‘connected water’ as a fraction of total terrestrial precipitation by ref. 4 based on remotely-sensed global deuterium mass balance. We note that these estimates (including our own) do not necessarily equal the magnitude of groundwater contribution to transpiration fluxes, due to the existence of strong vegetation ecophysiological controls on transpiration fluxes (e.g. stomatal regulation of transpiration rates). Like ref. 132 we note that field-based sap flow measurements of total tree water use would be needed to make such estimates.

Our finding that most plants have limited groundwater use may have several implications for land surface models (LSMs) designed for explicit representation of saturated zone water in climate-vegetation feedbacks^167^. Regional- to global-scale climate projections are informed in part by LSMs that describe root water uptake as a function of changes in soil moisture^168^. Furthermore, our findings may also have implications for the assessment of plant vulnerability to, and growth impairment after, drought stress at plant and ecosystem scales^18^,^169^.

## Methods

### Data compilation and treatment

We searched the published literature for water stable isotope papers in ecology and hydrology. The search initially returned 615 and 533 papers from ISI Web of Science and Scopus, respectively. Papers without xylem (i.e. plant stem) isotope measurements were excluded. After title/abstract screening and removal of duplicates, a total of 138 records remained and were included in the systematic review and meta-analysis. We used Comprehensive Meta-analysis (version 3, BioStat Software, Engelwood, NJ, USA) as the statistical platform for all meta-analysis and associated statistical tests. We defined “groundwater” following the operational definitions in the respective source papers, which included ‘water below permanent groundwater tables’ or ‘aquifers to springs’ and ‘groundwater reservoirs in bedrock cracks and fissures’. We described groundwater influence on vegetation by quantifying prevalence and magnitude. We defined prevalence as the number of samples out of a universe of plant samples reported to have groundwater contribution to xylem water. We defined magnitude as the proportion of groundwater contribution in xylem water mixture. We acknowledge that future work involving newly collected data should go beyond the necessarily loose definition of “groundwater” used here (as mandated by the original paper synthesis) and consider how bedrock groundwater^132^ and other shallower groundwater^108^,^109^ might impact plant water sourcing. To summarize prevalence and magnitude findings, we computed prevalence and magnitude point estimates using the following:


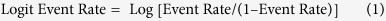



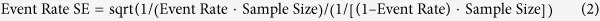


Confidence interval (CI) indicates the range within which the true mean (prevalence or magnitude) estimates will fall in 95% of all possible meta-analyses. 95% CI was computed using the following:


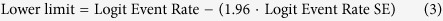



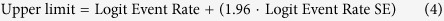


Prediction interval (PI) indicates the range within which 95% of all (prevalence or magnitude) estimates will fall in 95% of all meta-analyses. 95% PI was computed using the following:


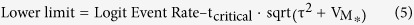



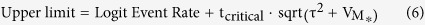


where t_critical_ is the two-tailed inverse of *t*-distribution at 95% interval, τ^2^ is the variance of true effects, and V_M*_ is variance of the summary effect estimated as the reciprocal of the sum of the weights.

Associated statistics, particularly *Q-value* and *I*^*2*^, were then calculated to supplement the description of the point and 95% CI estimates of prevalence and magnitude of groundwater influence. *Q-value* is a measure of weighted squared deviations, which quantifies true variation from observed variation^170^. That is, it provides a test of the null hypothesis that all studies in a particular analysis share a common prevalence (or as applicable, magnitude) estimate. This means that if the *Q-value* is equal to the degrees of freedom (*df; n *− 1) then all studies share the same prevalence or magnitude estimate. *I*^*2*^ is computed as ((Q − df)/Q) ∙ 100, and provides an estimate of the ratio of true heterogeneity to total variance across the observed prevalence (or magnitude) estimates^170^. That is, it indicates what proportion of the observed variance reflects differences in true effect sizes (i.e. prevalence or magnitude) rather than sampling error.

The ultimate goal of any meta-analysis is to estimate an overall effect size (in this case, prevalence or magnitude of groundwater influence) and associated CI. If the precision across all 138 published papers in our database were equal, we could readily compute for the simple mean of all prevalence (or magnitude) estimates. Since this is obviously not the case, we needed to compute a weighted mean by assigning weights to the studies. There are two computational model approaches in meta-analysis to achieve this: the fixed effect model and the random effects model. A fixed effect model would be appropriate if there was plausible reason to believe that all studies in the synthesis were functionally identical. This is generally not a valid assumption in any meta-analysis that synthesizes data from primary literature^170^. Thus, here we used the random effects model. In so doing, we weighted each study by the inverse of its original (within-studies) variance plus between-studies variance.

In the above equations, event rate with respect to prevalence of groundwater influence calculations refers to the number of samples out of a universe of xylem samples reported to have groundwater contribution in xylem. Two input variables were required: (1) number of samples reported to have groundwater contribution in xylem, and (2) total number of xylem samples. Event rate with respect to magnitude of groundwater influence calculations refers to the mean proportion of groundwater contribution in xylem. Two input variables were required: (1) mean proportion of groundwater contribution in xylem, and (2) total number of xylem samples. Our database consists of source papers (*n* = 138) that represent five main source identification or source apportionment approaches: (1) direct inference (*n* = 59); (2) IsoSource^155^, (*n* = 34); 2, 3-source mass balance (*n* = 32); Bayesian^153^,^154^ (*n* = 8); and custom author (“other”, *n* = 5). All data syntheses on prevalence and magnitude of groundwater influence were performed at a species level. Genus and other categorical variables (e.g. terrestrial biome, angiosperm/gymnosperm, source apportionment approach, etc.) were assigned accordingly to allow for associated statistical tests. To be conservative and avoid underestimation from a meta-analysis of IsoSource^155^ papers, we calculated the mean value of the reported maximum proportions. We also performed a meta-analysis using the mean value of reported minimum proportions and grand mean of reported mean values in IsoSource papers. Performing these resulted in estimates biased towards the lower end of the range reported in Fig. 2a. Where a source paper used any of the other four source identification/apportionment approaches, we calculated the mean value of all reported proportions. For studies with reported measurements at multiple time points (e.g. multiple dates through the year), we calculated the mean values of reported proportions at multiple time points and used the mean score in the calculation of prevalence and magnitude estimates^172^,^173^.

Ref. 9 showed that groundwater dependent ecosystems span a wide range, from wettest to driest environments. This implies that controls on groundwater use by vegetation are manifold, possibly a combination of a host of factors that vary from one site and/or species to the next^160^,^174^,^175^,^176^. For example, ref. 160 showed that in an extremely arid setting (annual precipitation ~35 mm, potential evaporation ~2600 mm), plant physiological parameters (e.g. leaf-specific hydraulic conductance and leaf water relations of phreatophytes) were related to groundwater depth. By extension, rooting depth and rooting zone water storage were also related to groundwater depth. To achieve a multivariate characterization of the magnitude of groundwater influence, we supplemented the primary information available from source papers with site-specific datasets: rooting depth^177^, rooting zone water storage amount^178^, water table depth^179^, potential evapotranspiration and aridity index^180^, precipitation amount^166^, and streamflow amount^165^. We also derived actual evapotranspiration (AET) as the difference between precipitation and streamflow^180^. This resulted in a table with the aforementioned variables as data columns in addition to the mean magnitude estimates of groundwater contribution from the random effects model in meta-analysis. We then used a batch algorithm and a locally weighted linear smoother (self-organizing maps clustering technique^171^) to explore the structure of the meta-analysis database. Like classical *k*-means clustering, self-organizing maps (SOMs) technique performs an iterative alternating fitting process that results in the formation of a number of clusters.

Each cluster is a set of points that share similar values across a number of variables. Unlike the classical *k*-means clustering, SOMs form clusters in a particular layout on a grid. This results in the formation of clusters with points that are near each other in the SOM grid as well as in multivariate space. The SOM technique was an appropriate treatment of our data because of the wide range of variability in the magnitude estimates of groundwater influence. The SOM was implemented first by using principal components analysis thereby determining the two directions that captured the most variation in the data. It then laid out a grid in the principal component space thereby forming clusters back in the original space of the variables. The same clustering principle and estimation of means as in *k*-means approach was then used in assigning clusters. This analysis resulted in the identification of seven clusters shown in Fig. 3. The inset in Fig. 3 shows an intuitive outcome of the SOM approach used here wherein the magnitude of groundwater influence increases with aridity (i.e. inversely proportional to aridity index).

### Publication bias

Any meta-analysis is prone to publication bias (i.e. the file-drawer problem^182^). This is a function, by and large, of the inherent propensity of many journals to not publish negative results. A meta-analysis such as this contribution, therefore, may not be immune to such publication bias in the most fundamental sense. Nevertheless, here we addressed this issue in two phases: (Phase 1) structural intervention to mitigate against bias (before data synthesis and meta-analysis); (Phase 2) statistical assessment of bias (after data synthesis and meta-analysis).

Phase 1 was achieved by not discriminating against studies performed in any particular physiographic setting. For example, rather than synthesizing information exclusively from source papers in settings reported to have groundwater influence, we synthesized information from source papers across all habitat types. Phase 2 was achieved by performing graphical and statistical analysis to assess bias. Extended Data Fig. 1 shows a funnel plot of standard error by logit event rate. Large studies tend to cluster near the mean effect size and toward the top of the graph. Smaller studies tend to be more dispersed (i.e. greater sampling variation in prevalence estimates) and toward the bottom of the graph. In the absence of publication bias studies would be distributed symmetrically about the combined effect size. The absence of publication bias in our study is confirmed as shown in Extended Data Fig. 1. We also employed three statistical approaches to assess bias: Classic *Fail-safe N*^183^, Begg and Mazumdar Rank Correlation Test^170^,^184^, and Duval and Tweedie’s Trim and Fill^185^. Classic *Fail-safe N* yielded a *z*-value of −5.37594, a corresponding 2-tailed *P* < 0.0001, and a *Fail-safe N* of 901. This means that we would need to locate and include 901 ‘null’ studies in order for the combined 2-tailed *P*-value to exceed 0.050, and therefore nullify our results. Given that the initial number of returned studies from our database search was between 533 and 615, here we rule out bias based on this approach.

**Figure 1:**
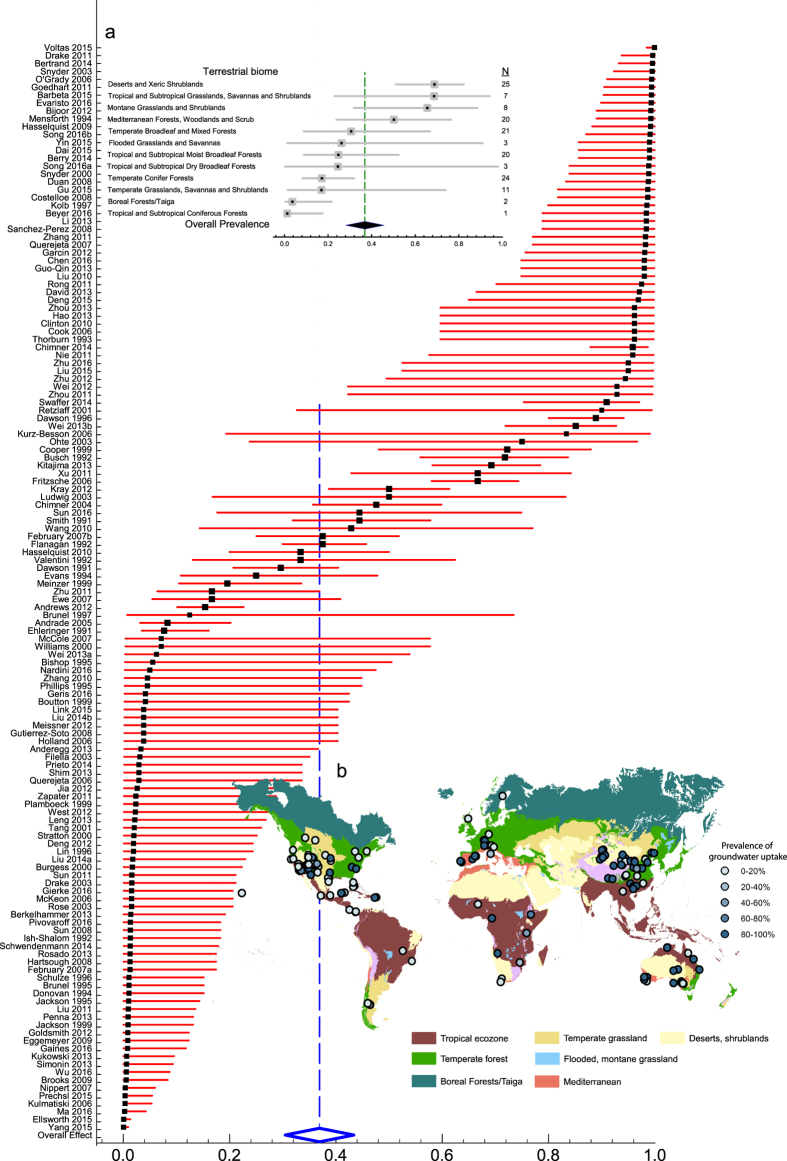
Prevalence of groundwater influence (x-axis: 0 corresponds to no groundwater influence, greater than 0 to groundwater influence). **Main plot**, prevalence estimates grouped by source paper (first author-year format). Filled black squares are prevalence point estimates, error bars are 95% confidence intervals (CI, red horizontal lines). Open diamond represents overall prevalence value and its 95% CI is represented by the width of diamond. (**a**), prevalence estimates grouped by terrestrial biome with N representing corresponding number of sites; (**b**), Map of prevalence estimates in 162 sites in the global meta-analysis database. Terrestrial biomes delineated by The Nature Conservancy http://www.nature.org. Map was generated using ArcMap 10.2 (http://services.arcgisonline.com/arcgis/rest/services/World_Street_Map/MapServer).

**Figure 2:**
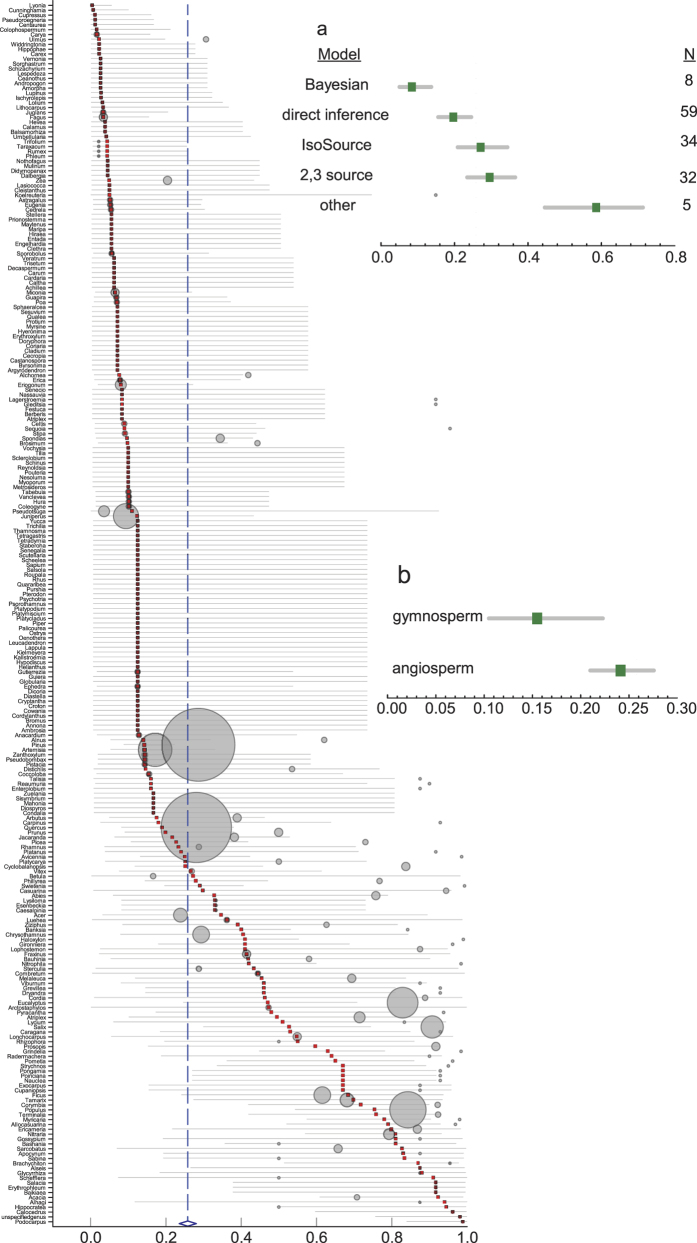
Magnitude of groundwater influence. **Forest plot**, magnitude estimates grouped by genus. Filled squares are magnitude point estimates, error bars are 95% confidence intervals (CI). Open blue diamond represents overall magnitude value and its 95% CI is represented by the width of diamond. **Bubble plot**, mean prevalence point estimates grouped by genus. Bubble size represents the corresponding number of studies (i.e. source papers) about one particular plant genus (e.g. Pinus and Quercus with N = 26 and 25, respectively). (**a**), magnitude estimates grouped by source identification/apportionment approach with N representing corresponding number of source papers; (**b**), magnitude estimates grouped by angiosperm and gymnosperm.

**Figure 3:**
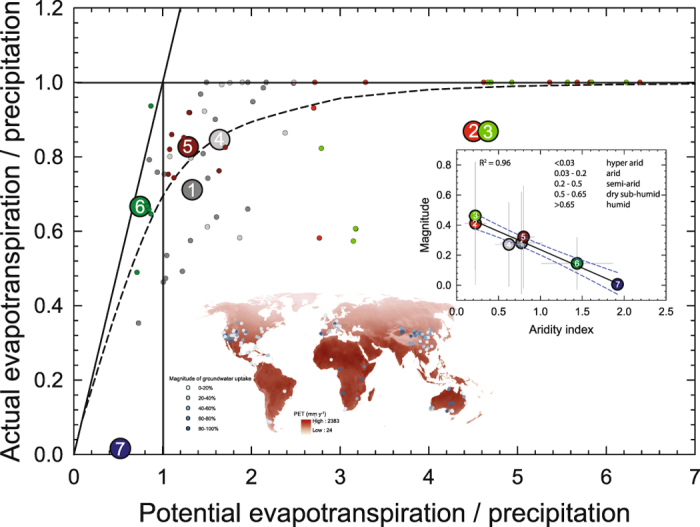
Cluster analysis (self-organizing maps) plot in evaporative index (actual evapotranspiration/precipitation^166^) as a function of dryness index (potential evapotranspiration 180**/precipitation).** Actual evapotranspiration (AET) was calculated as the difference between precipitation and streamflow^181^. Small dots are sites in cluster analysis with colors representing corresponding cluster assignments. **Inset**, magnitude estimates of cluster assignments as a function of aridity index. Error bars are 1 SD. Also shown is the generalized climate classification scheme for global aridity index^180^ and map of magnitude of groundwater influence over potential evapotranspiration (PET) layer (mm y^−1^) annual average 1950–2000^180^. Map was generated using ArcMap 10.2 (http://services.arcgisonline.com/arcgis/rest/services/World_Street_Map/MapServer).

The Begg and Mazumdar Rank Correlation Test^170^,^184^ computes the rank order correlation between the prevalence and magnitude estimates and the standard error (driven primarily by sample size). They suggest that a significant correlation points to the existence of bias. Based on this approach the 2-tailed *P*-value was 0.35739, which means that we could rule out the existence of bias. Finally, the Duval and Tweedie Trim and Fill^185^ suggests a way to determine where the missing studies were likely to fall if at all bias existed, add them to the database and then reanalyze the overall prevalence estimates. Using this approach, the prevalence estimate and 95% CI is 0.36915 (0.28443, 0.46277), which remained unchanged from the original (Fig. 1). The unchanged values suggest, and confirm earlier indications of, the absence of publication bias. We note the approaches that we employed in Phase 1 (literature search process) and Phase 2 (assessment of publication bias) are in compliance with “state of the art” and “state of the practice” in meta-analyses, particularly the PRISMA (Preferred Reporting Items for Systematic reviews and Meta-Analyses) guidelines^186^.

### Sensitivity analysis

To be able to test the robustness of the overall estimate of groundwater influence [23 (95% CI, 20–26%)], we performed a sensitivity analysis by removing one study at a time and recalculating the effect of this “removal” on the overall estimate. This was performed for all 138 source papers and at each corresponding genus (*n* = 251) and species (*n* = 414). Results of the sensitivity analysis showed markedly minimal effect on the overall estimate, with mean point estimates ranging between 22.68% and 23.36%; 95% lower limit ranging between 19.83% and 20.59%; 95% upper limit ranging between 25.81% and 26.38%.

### Isotope systems

When the analysis is grouped by isotope system used, we note that source papers that used δ^2^H (*n* = 35), δ^18^O (*n* = 31), or δ^2^H-δ^18^O (*n* = 72) isotope systems yield prevalence and magnitude estimates that may be different from each other. With respect to prevalence estimates, δ^2^H and δ^2^H- δ^18^O source papers tended to be similar (not statistically different) to each other: 32% (95% CI, 20–46%) and 31% (95% CI, 17–50%), respectively. δ^18^O tended to overestimate prevalence at 55% (95% CI, 38–72%). With respect to magnitude estimates, δ^2^H and δ^18^O source papers tended to be comparable (not statistically different) with each other: 32% (95% CI, 23–41%) and 44% (95% CI, 33–55%), respectively. δ^2^H-δ^18^O tended to underestimate magnitude at 15% (95% CI, 11–20%).

### Taxonomic and biome effects on groundwater use

Approximately 93% of taxonomic information in our database were referenced from National Center for Biotechnology Information (NCBI http://www.ncbi.nlm.nih.gov/taxonomy).

## Additional Information

**How to cite this article**: Evaristo, J. and McDonnell, J. J. Prevalence and magnitude of groundwater use by vegetation: a global stable isotope meta-analysis. *Sci. Rep.* **7**, 44110; doi: 10.1038/srep44110 (2017).

**Publisher's note:** Springer Nature remains neutral with regard to jurisdictional claims in published maps and institutional affiliations.

## Supplementary Material

Supplementary Figure 1

Supplementary Dataset 1
