# Supplementary figures and images for: Prevalence and magnitude of groundwater use by vegetation: a global stable isotope meta-analysis
Source: Sci Rep. 2017 Mar 10;7:44110. doi: 10.1038/srep44110 (PMC5345103; doi:10.1038/srep44110)

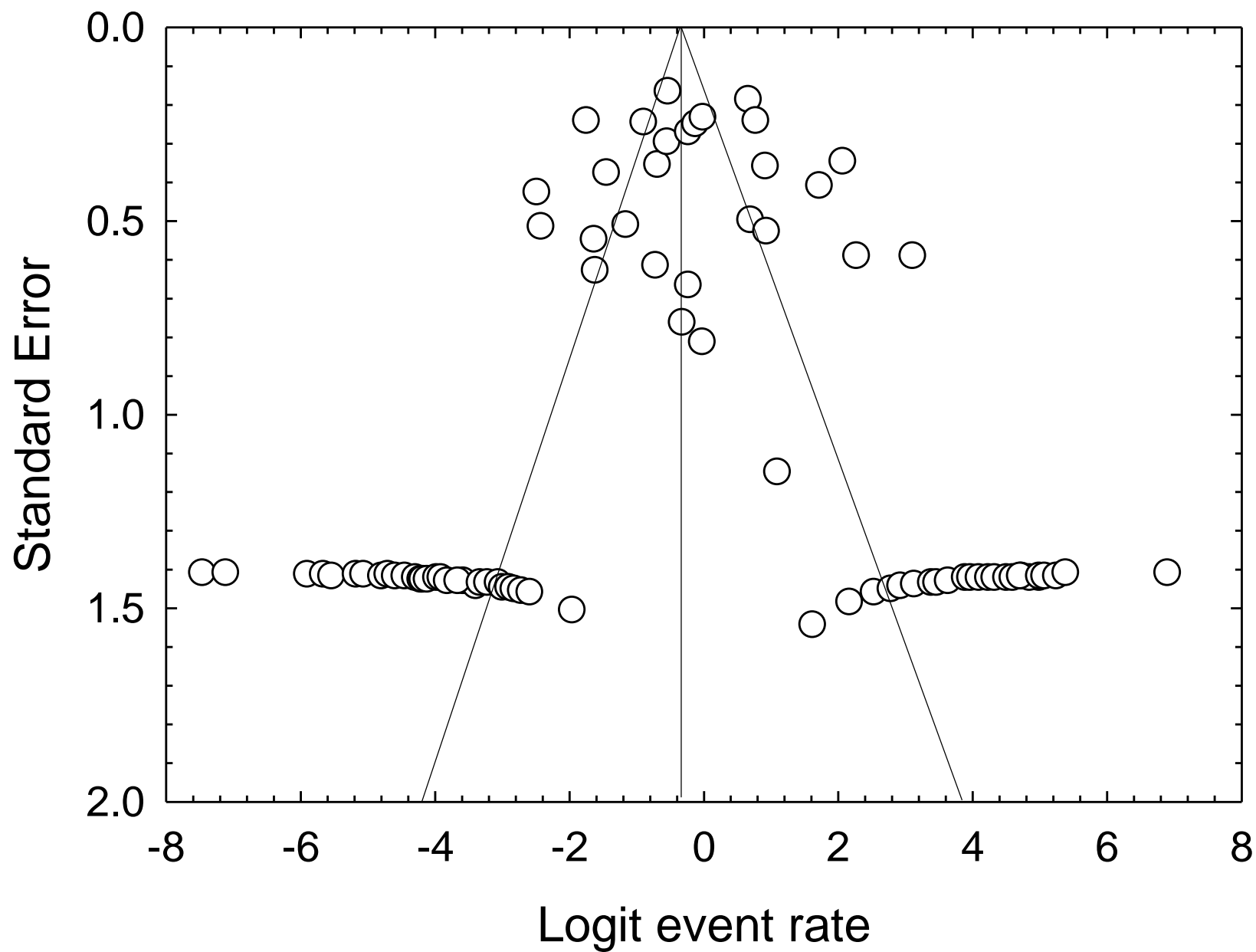

Supplement: Supplementary Figure 1 [file srep44110-s1.pdf]
